# *rac*-4,8-Divinyl­bicyclo­[3.3.1]nonane-2,6-dione

**DOI:** 10.1107/S1600536809018443

**Published:** 2009-05-20

**Authors:** Edvinas Orentas, Ola F. Wendt, Kenneth Wärnmark, Eugenijus Butkus, Carl-Johan Wallentin

**Affiliations:** a Department of Organic Chemistry, Vilnius University, Naugarduko 24, LT-03225 Vilnius, Lithuania; b Organic Chemistry, Department of Chemistry, Lund University, PO Box 124, S-221 00 Lund, Sweden

## Abstract

The title compound, C_13_H_16_O_2_, is a chiral bicyclic structure composed of two fused cyclo­hexa­ne rings possessing both boat and chair conformations. The mol­ecules are packed in enantio­pure columns which are pairwise linked forming an overall racemic solid; within the column pairs the packing is governed by weak dipole–dipole inter­actions stemming from stacked carbonyl functionalities (CO_centroid_–CO_centroid_ distance = 3.290 Å).

## Related literature

For related structures, see: Orentas *et al.* (2007 ▶); Quast *et al.* (1994 ▶, 1999 ▶); Wallentin *et al.* (2009 ▶). For a general background to non-covalent inter­actions, see: Desiraju & Steiner (1999 ▶); Aakeröy (1997 ▶), and references therein.
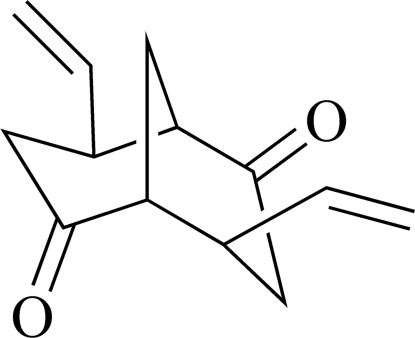


## Experimental

### 

#### Crystal data

C_13_H_16_O_2_
*M* *_r_* = 204.26
Orthorhombic, 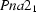

*a* = 20.4254 (11) Å
*b* = 8.8913 (6) Å
*c* = 6.2570 (4) Å
*V* = 1136.32 (12) Å^3^
*Z* = 4
Mo *K*α radiation
μ = 0.08 mm^−1^
*T* = 293 K
0.3 × 0.05 × 0.03 mm

#### Data collection

Oxford Diffraction Xcalibur diffractometer
Absorption correction: multi-scan (*CrysAlis RED*; Oxford Diffraction, 2006 ▶) *T*_min_ = 0.827, *T*_max_ = 1.000 (expected range = 0.825–0.998)
8068 measured reflections
1363 independent reflections
811 reflections with *I* > 2σ(*I*)
*R*_int_ = 0.031

#### Refinement

*R*[*F* ^2^ > 2σ(*F* ^2^)] = 0.044
*wR*(*F* ^2^) = 0.110
*S* = 1.02
1363 reflections
136 parameters
1 restraint
H-atom parameters constrained
Δρ_max_ = 0.32 e Å^−3^
Δρ_min_ = −0.19 e Å^−3^

### 

Data collection: *CrysAlis CCD* (Oxford Diffraction, 2006 ▶); cell refinement: *CrysAlis RED* (Oxford Diffraction, 2006 ▶); data reduction: *CrysAlis RED*; program(s) used to solve structure: *SIR97* (Altomare *et al.*, 1999 ▶); program(s) used to refine structure: *SHELXL97* (Sheldrick, 2008 ▶); molecular graphics: *DIAMOND* (Brandenburg, 2000 ▶); software used to prepare material for publication: *WinGX* (Farrugia, 1999 ▶).

## Supplementary Material

Crystal structure: contains datablocks global, I. DOI: 10.1107/S1600536809018443/ng2580sup1.cif

Structure factors: contains datablocks I. DOI: 10.1107/S1600536809018443/ng2580Isup2.hkl

Additional supplementary materials:  crystallographic information; 3D view; checkCIF report

## Figures and Tables

**Fig. 1.**
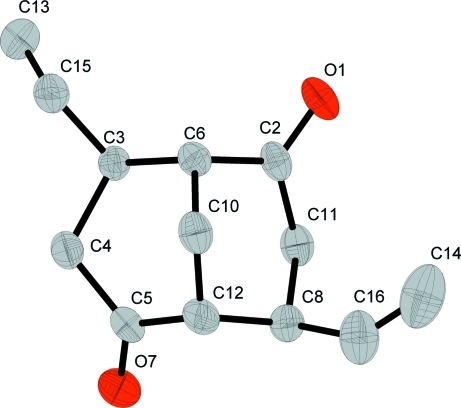
The molecular structure of the title compound with atom labels and 30% probability displacement ellipsoids.

**Fig. 2.**
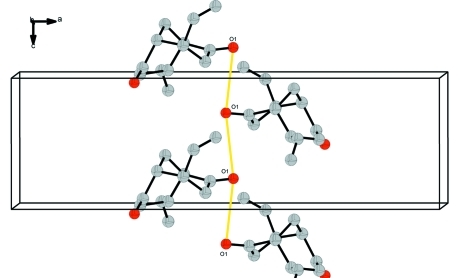
A partial packing diagram of the title compound displaying the weak interactions that govern the formation of column pairs. Dipol-dipol interactions are visualized as yellow lines connecting the carbonyl O atoms.

## supplementary crystallographic information

### Comment

The hydrocarbon backbone of the title compound is a common motif in many biologically active compounds and its unique molecular shape has been utilized in the construction of different types of supramolecular architectures and inclusion complexes. Diols from this category of structures has with great success been exploited within the field of crystal engineering. The title compound was obtained in the synthesis of a series of C_2_ -symmetrically derivatized bicyclo[3.3.1]nonane-2,6-diones as a part of an ongoing project with the aim to study various supramolecular features of this class of compounds. The chiral bicyclic structure is composed of two merged cyclohexanes possessing both boat and chair conformations similar to the previously reported phenyl-substituted bicyclo[3.3.1]nonane-2,6-dione (Quast *et al.*, 1999). The molecules are packed in column pairs which propagate in a unidirectional manner along the *c* axis. The column pairs are homochiral and generated by a two fold screw axis. The glide plane generates an over-all racemic structure comprised of parallel columns with alternating absolute stereochemistry. The formation of column pairs is governed by dipole-dipole interactions stemming from stacked carbonyl functionalities: centroid C2O1···centroid C2O1, 3.290 Å.

### Experimental

A solution of LiCl in THF ( 0.5M, 12.5 mL, 6.25 mmol) was added to a round-bottom flask charged with CuCN (0.272 g, 3.04 mmol) and the mixture was stirred under argon at room temperature until all solid dissolved. The solution was cooled down to -30 °C and a solution of vinyl magnesium bromide in THF (0.7 M, 4.34 mL, 3.04 mmol) was added dropwise. The resulting dark brown mixture was warmed to -20 °C and stirred at this temperature for 30 minutes and then cooled to -78 °C. A solution of bicyclo[3.3.1]nona-3,7-diene-2,6-dione (0.15g, 1.01 mmol) (Orentas *et al.*, 2007) and trimethylsilylchloride (0.33 g, 3.04 mmol) in THF (3 mL) was added dropwise. The reaction mixture was stirred until the temperature reached -20 °C. The reaction was quenched with 10% HCl solution (20 mL) and stirred at room temperature until the intermediate silylenol ether was hydrolyzed (monitored by TLC). The mixture was diluted with water and extracted with EtOAc (3 x 20 mL). The combined organic phase was dried over Na_2_SO_4_ and evaporated to dryness. The residue was purified by flash chromatography (10% ethyl acetate/petroleum ether) to afford the title compound as a coluorless solid in 70 % yield (144.4 mg). The product was recrystallised from petroleum ether to give colourless crystals suitable for X-ray diffraction analysis; m.p. 65 °C; FTIR (KBr) 1700 (C=O) cm^-1^; ^1^H NMR (300 MHz, CDCl_3_) δ 5.83 (ddd, J_1_=17.0 Hz, J_2_=10.4 Hz, J_3_=6.0 Hz, 1H), 5.17 (dd, J_1_=10.4 Hz, J_2_=1.6 Hz, 1H), 5.10 (dd, J_1_=17.0 Hz,J_2_=1.6 Hz, 1H), 2.93-2.83 (m, 2H), 2.62-2.53 (m, 6H), 2.19-2.14 (m, 2H); ^13^C NMR (75 MHz, CDCl_3_) δ 211.86, 139.34, 116.01, 48.10, 39.46, 22.81; Anal. calcd for C_13_H_16_O_2_: C, 76.44; H, 7.90. Found: C, 76.57; H, 8.02.

### Refinement

The H atoms were positioned geometrically and treated as riding on their parent atoms with C–H distances of 0.93–0.97 Å and *U*_iso_(H) = 1.2*U*_eq_ - 1.5*U*_eq_. Equivalent reflections including Friedel pairs were merged prior to the final refinement.

### Crystal data

**Table:**

C_13_H_16_O_2_	*F*(000) = 440
*M**_r_* = 204.26	*D*_x_ = 1.194 Mg m^−^^3^
Orthorhombic, *P**n**a*2_1_	Mo *K*α radiation, λ = 0.71073 Å
Hall symbol: P 2c -2n	Cell parameters from 2363 reflections
*a* = 20.4254 (11) Å	θ = 2.3–33.1°
*b* = 8.8913 (6) Å	µ = 0.08 mm^−^^1^
*c* = 6.2570 (4) Å	*T* = 293 K
*V* = 1136.32 (12) Å^3^	Prism, colourless
*Z* = 4	0.3 × 0.05 × 0.03 mm

### Data collection

**Table:**

Oxford Diffraction Xcalibur diffractometer	1363 independent reflections
Radiation source: Enhance (Mo) X-ray Source	811 reflections with *I* > 2σ(*I*)
graphite	*R*_int_ = 0.031
ω scans	θ_max_ = 27.1°, θ_min_ = 3.0°
Absorption correction: multi-scan (*CrysAlis RED*; Oxford Diffraction, 2006)	*h* = −24→26
*T*_min_ = 0.827, *T*_max_ = 1.000	*k* = −11→11
8068 measured reflections	*l* = −5→8

### Refinement

**Table:**

Refinement on *F*^2^	1 restraint
Least-squares matrix: full	H-atom parameters constrained
*R*[*F*^2^ > 2σ(*F*^2^)] = 0.044	*w* = 1/[σ^2^(*F*_o_^2^) + (0.06*P*)^2^] where *P* = (*F*_o_^2^ + 2*F*_c_^2^)/3
*wR*(*F*^2^) = 0.110	(Δ/σ)_max_ < 0.001
*S* = 1.02	Δρ_max_ = 0.32 e Å^−^^3^
1363 reflections	Δρ_min_ = −0.19 e Å^−^^3^
136 parameters	

### Special details

**Table:**

Experimental. The intensity data were collected on a Oxford Xcalibur 3 CCD diffractometer using an exposure time of 20 s/frame. A total of 552 frames were collected with a frame width of 0.5° covering up to θ = 27.09° with 99.9% completeness accomplished. The highest difference peak in the Fourier map is located 0.85 Å from H16.
Geometry. All e.s.d.'s (except the e.s.d. in the dihedral angle between two l.s. planes) are estimated using the full covariance matrix. The cell e.s.d.'s are taken into account individually in the estimation of e.s.d.'s in distances, angles and torsion angles; correlations between e.s.d.'s in cell parameters are only used when they are defined by crystal symmetry. An approximate (isotropic) treatment of cell e.s.d.'s is used for estimating e.s.d.'s involving l.s. planes.

### Fractional atomic coordinates and isotropic or equivalent isotropic displacement parameters (Å^2^)

**Table:**

	*x*	*y*	*z*	*U*_iso_*/*U*_eq_	
C6	0.60714 (11)	0.5148 (3)	0.2569 (5)	0.0501 (7)	
H6	0.5959	0.6085	0.1829	0.060*	
O1	0.49120 (9)	0.4888 (3)	0.2890 (4)	0.0684 (6)	
C2	0.54437 (13)	0.4308 (3)	0.3065 (4)	0.0522 (7)	
C3	0.64496 (12)	0.5550 (3)	0.4657 (5)	0.0490 (7)	
H3	0.6162	0.5334	0.5872	0.059*	
C4	0.70813 (12)	0.4602 (3)	0.4926 (5)	0.0587 (8)	
H4A	0.7424	0.5028	0.4039	0.070*	
H4B	0.7225	0.4661	0.6402	0.070*	
C5	0.69861 (13)	0.2986 (4)	0.4334 (6)	0.0635 (8)	
O7	0.71657 (11)	0.1941 (3)	0.5432 (5)	0.0990 (10)	
C8	0.60166 (13)	0.1778 (3)	0.2573 (6)	0.0609 (8)	
H8	0.6137	0.0912	0.3459	0.073*	
C10	0.64888 (14)	0.4191 (3)	0.1080 (5)	0.0592 (8)	
H10A	0.6891	0.4718	0.0730	0.071*	
H10B	0.6253	0.3995	−0.0236	0.071*	
C11	0.55214 (12)	0.2721 (3)	0.3865 (5)	0.0571 (8)	
H11A	0.5099	0.2226	0.3821	0.069*	
H11B	0.5662	0.2752	0.5345	0.069*	
C12	0.66464 (13)	0.2711 (3)	0.2209 (5)	0.0607 (8)	
H12	0.6942	0.2129	0.1294	0.073*	
C15	0.66318 (14)	0.7178 (3)	0.4753 (6)	0.0652 (9)	
H15	0.6861	0.7569	0.3595	0.078*	
C16	0.5761 (2)	0.1174 (5)	0.0507 (7)	0.0924 (12)	
H16	0.6053	0.0540	−0.0195	0.111*	
C13	0.64974 (18)	0.8101 (4)	0.6321 (7)	0.0892 (12)	
H13A	0.6269	0.7758	0.7511	0.107*	
H13B	0.6630	0.9100	0.6247	0.107*	
C14	0.5247 (3)	0.1352 (5)	−0.0434 (7)	0.1147 (16)	
H14A	0.4925	0.1969	0.0145	0.138*	
H14B	0.5175	0.0872	−0.1734	0.138*	

### Atomic displacement parameters (Å^2^)

**Table:**

	*U*^11^	*U*^22^	*U*^33^	*U*^12^	*U*^13^	*U*^23^
C6	0.0477 (14)	0.0546 (15)	0.0480 (16)	0.0009 (13)	−0.0027 (14)	0.0079 (14)
O1	0.0420 (10)	0.1030 (15)	0.0603 (12)	0.0124 (11)	−0.0049 (10)	−0.0058 (11)
C2	0.0405 (15)	0.076 (2)	0.0406 (16)	0.0009 (13)	−0.0050 (12)	−0.0052 (14)
C3	0.0443 (14)	0.0523 (15)	0.0504 (16)	−0.0024 (13)	0.0013 (12)	−0.0010 (14)
C4	0.0489 (16)	0.0657 (18)	0.061 (2)	−0.0011 (14)	−0.0076 (14)	−0.0050 (16)
C5	0.0429 (15)	0.067 (2)	0.080 (2)	0.0088 (15)	−0.0110 (16)	−0.0021 (18)
O7	0.0928 (17)	0.0737 (15)	0.131 (2)	0.0123 (12)	−0.0493 (19)	0.0152 (17)
C8	0.0605 (17)	0.0593 (17)	0.0628 (18)	−0.0035 (14)	−0.0097 (18)	−0.0021 (17)
C10	0.0461 (15)	0.081 (2)	0.0507 (18)	−0.0096 (15)	0.0074 (14)	−0.0017 (17)
C11	0.0464 (15)	0.072 (2)	0.0531 (16)	−0.0107 (13)	0.0031 (13)	0.0032 (16)
C12	0.0449 (15)	0.0669 (18)	0.0702 (19)	0.0079 (14)	0.0060 (15)	−0.0096 (18)
C15	0.0522 (16)	0.064 (2)	0.079 (2)	−0.0059 (15)	−0.0006 (16)	0.0030 (19)
C16	0.074 (2)	0.115 (3)	0.088 (3)	−0.022 (2)	0.005 (3)	−0.016 (3)
C13	0.080 (2)	0.071 (2)	0.116 (3)	−0.0003 (19)	−0.017 (2)	−0.022 (2)
C14	0.140 (4)	0.120 (3)	0.084 (3)	−0.049 (3)	−0.011 (3)	0.002 (3)

### Geometric parameters (Å, °)

**Table:**

C6—C2	1.516 (4)	C8—C12	1.548 (4)
C6—C10	1.522 (4)	C8—H8	0.9800
C6—C3	1.560 (4)	C10—C12	1.528 (4)
C6—H6	0.9800	C10—H10A	0.9700
O1—C2	1.207 (3)	C10—H10B	0.9700
C2—C11	1.506 (4)	C11—H11A	0.9700
C3—C15	1.496 (4)	C11—H11B	0.9700
C3—C4	1.550 (4)	C12—H12	0.9800
C3—H3	0.9800	C15—C13	1.308 (4)
C4—C5	1.497 (4)	C15—H15	0.9300
C4—H4A	0.9700	C16—C14	1.215 (5)
C4—H4B	0.9700	C16—H16	0.9300
C5—O7	1.212 (4)	C13—H13A	0.9300
C5—C12	1.519 (5)	C13—H13B	0.9300
C8—C16	1.494 (5)	C14—H14A	0.9300
C8—C11	1.543 (4)	C14—H14B	0.9300
			
C2—C6—C10	108.9 (2)	C6—C10—C12	108.4 (2)
C2—C6—C3	111.1 (2)	C6—C10—H10A	110.0
C10—C6—C3	111.3 (2)	C12—C10—H10A	110.0
C2—C6—H6	108.5	C6—C10—H10B	110.0
C10—C6—H6	108.5	C12—C10—H10B	110.0
C3—C6—H6	108.5	H10A—C10—H10B	108.4
O1—C2—C11	121.7 (2)	C2—C11—C8	113.8 (2)
O1—C2—C6	122.1 (3)	C2—C11—H11A	108.8
C11—C2—C6	116.1 (2)	C8—C11—H11A	108.8
C15—C3—C4	108.3 (2)	C2—C11—H11B	108.8
C15—C3—C6	112.3 (2)	C8—C11—H11B	108.8
C4—C3—C6	112.2 (2)	H11A—C11—H11B	107.7
C15—C3—H3	107.9	C5—C12—C10	111.3 (2)
C4—C3—H3	107.9	C5—C12—C8	109.7 (3)
C6—C3—H3	107.9	C10—C12—C8	110.8 (2)
C5—C4—C3	112.8 (2)	C5—C12—H12	108.3
C5—C4—H4A	109.0	C10—C12—H12	108.3
C3—C4—H4A	109.0	C8—C12—H12	108.3
C5—C4—H4B	109.0	C13—C15—C3	125.8 (3)
C3—C4—H4B	109.0	C13—C15—H15	117.1
H4A—C4—H4B	107.8	C3—C15—H15	117.1
O7—C5—C4	123.8 (3)	C14—C16—C8	132.4 (4)
O7—C5—C12	120.8 (3)	C14—C16—H16	113.8
C4—C5—C12	115.5 (3)	C8—C16—H16	113.8
C16—C8—C11	114.8 (3)	C15—C13—H13A	120.0
C16—C8—C12	110.8 (3)	C15—C13—H13B	120.0
C11—C8—C12	109.3 (2)	H13A—C13—H13B	120.0
C16—C8—H8	107.2	C16—C14—H14A	120.0
C11—C8—H8	107.2	C16—C14—H14B	120.0
C12—C8—H8	107.2	H14A—C14—H14B	120.0
			
C10—C6—C2—O1	−130.0 (3)	C16—C8—C11—C2	−79.0 (3)
C3—C6—C2—O1	107.1 (3)	C12—C8—C11—C2	46.2 (3)
C10—C6—C2—C11	52.1 (3)	O7—C5—C12—C10	−177.9 (3)
C3—C6—C2—C11	−70.9 (3)	C4—C5—C12—C10	1.6 (3)
C2—C6—C3—C15	−128.9 (3)	O7—C5—C12—C8	59.1 (4)
C10—C6—C3—C15	109.5 (3)	C4—C5—C12—C8	−121.3 (3)
C2—C6—C3—C4	108.7 (2)	C6—C10—C12—C5	−56.8 (3)
C10—C6—C3—C4	−12.8 (3)	C6—C10—C12—C8	65.5 (3)
C15—C3—C4—C5	−166.1 (3)	C16—C8—C12—C5	−166.2 (3)
C6—C3—C4—C5	−41.6 (3)	C11—C8—C12—C5	66.2 (3)
C3—C4—C5—O7	−132.2 (3)	C16—C8—C12—C10	70.6 (3)
C3—C4—C5—C12	48.3 (3)	C11—C8—C12—C10	−57.0 (3)
C2—C6—C10—C12	−60.4 (3)	C4—C3—C15—C13	−109.0 (3)
C3—C6—C10—C12	62.4 (3)	C6—C3—C15—C13	126.5 (3)
O1—C2—C11—C8	136.2 (3)	C11—C8—C16—C14	5.1 (6)
C6—C2—C11—C8	−45.8 (3)	C12—C8—C16—C14	−119.3 (5)

## References

[bb1] Aakeröy, C. B. (1997). *Acta Cryst.* B**53**, 569–586.

[bb2] Altomare, A., Burla, M. C., Camalli, M., Cascarano, G. L., Giacovazzo, C., Guagliardi, A., Moliterni, A. G. G., Polidori, G. & Spagna, R. (1999). *J. Appl. Cryst.* **32**, 115–119.

[bb3] Brandenburg, K. (2000). *DIAMOND* Crystal Impact GbR, Bonn, Germany.

[bb4] Desiraju, G. R. & Steiner, T. (1999). In *The Weak Hydrogen Bond* New York: Oxford University Press Inc.

[bb5] Farrugia, L. J. (1999). *J. Appl. Cryst.* **32**, 837–838.

[bb6] Orentas, E., Bagdziunas, G., Berg, U., Zilinskas, A. & Butkus, E. (2007). *Eur. J. Org. Chem.* pp. 4251–4256.

[bb7] Oxford Diffraction (2006). *CrysAlis CCD* and *CrysAlis RED* Oxford Diffraction Ltd, Abingdon, Oxfordshire, England.

[bb8] Quast, H., Becker, C., Geibler, E., Knoll, K., Peters, E.-M., Peters, K. & von Schnering, H. G. (1994). *Liebigs Ann. Chem.* pp. 109–120.

[bb9] Quast, H., Seefelder, M., Peters, E.-M. & Peters, K. (1999). *Eur. J. Org. Chem.* pp. 1811–1823.

[bb10] Sheldrick, G. M. (2008). *Acta Cryst.* A**64**, 112–122. 10.1107/S0108767307043930 18156677

[bb11] Wallentin, C.-J., Orentas, E., Johnson, M. T., Butkus, E., Wendt, O. F., Öhrström, L. & Wärnmark, K. (2009). *CrystEngComm* Submitted.

